# Episodes of extreme international capital inflows in emerging and developing economies: The role of global economic policy uncertainty

**DOI:** 10.1371/journal.pone.0275249

**Published:** 2022-09-28

**Authors:** Xiaoqing An, Bingyan Wu, Alisher Tohirovich Dedahanov, Wei Sun

**Affiliations:** 1 School of Economics, Jinan University, Guangzhou City, Guangdong Province, China; 2 School of Business, Akfa University, Tashkent, Uzbekistan; 3 School of Economics, Anyang Normal University, Anyang City, Henan Province, China; University of Sargodha, PAKISTAN

## Abstract

Based on annual data for 31 emerging and developing economies in the period 2000–2020, this paper explores the impact of global economic policy uncertainty on episodes of extreme international capital inflows. Following previous researches, we identify episodes of surges and stops. The results show that the global economic policy uncertainty can significantly increase the probability of surges and decrease the probability of stops in emerging and developing economies. The heterogeneity tests show that these effects vary with economic growth, financial development, the degree of economic globalization and global liquidity. Above effects are significant in the groups with higher economic growth, higher financial development, higher economic globalization and higher global liquidity. Further analyses show that as global economic policy uncertainty rises, its impact on surges and stops gradually declines. In addition, global economic policy uncertainty has a significant negative effect on the surge in advanced economies, which could confirm the above conclusion to a certain extent.

## 1. Introduction

Since the 1990s, economic globalization has gradually deepened so that the outbreak of any crisis has invariably made the emerging and developing economies suffer from the impact of international capital inflows and outflows frequently. Undeniably, episodes of surges can effectively alleviate the contradiction between capital supply and demand in emerging and developing economies. Surges can significantly promote economic growth, but they also lay a hidden danger for financial stability. As emerging and developing economies rely on foreign trade and international investment, episodes of stops are very likely to trigger a financial crisis and bring severe challenges to prudent macro management. Therefore, the management of international capital flows in emerging markets has drawn close attention from government departments and academic circles.

Under the strategic deployment of “building a new development pattern of domestic and international dual circulation”, China will further accelerate the pace of opening-up in the future which will broaden the channels of international capital flows [1]. As the largest emerging and developing economy in the world, China will inevitably bear the brunt of the impact of extreme movements in capital inflows. Thus, it is the top priority to explore the corresponding causes. At present, relevant studies on the causes mainly focus on the traditional push factors and pull factors. For emerging and developing economies, episodes of extreme international capital flows are mainly affected by the international economic environment [2]. Global risk index can decrease the probability of surges and increase the probability of stops, while economic growth and global liquidity have the opposite effect on the probability of surges and stops [1, 3, 4], and the dollar cycle will simultaneously reduce their possibilities [5]. In addition, scholars’ studies have further focused on sudden stops. For example, some scholars discuss the role of financial friction in the formation mechanism of sudden stops from the perspective of incomplete financial markets [6, 7]. Other scholars subdivide the sudden stops into inflow-driven and outflow-driven types to analyze the influence of openness, interest rate and economic growth on them [8, 9]. In addition, attention has also been paid to whether the adjustment of sovereign credit rating is helpful for reducing the probability of stops [10]. Driven by global economic integration, the influence of capital account liberalization on sudden stops has received more and more attention. However, early studies held that capital account liberalization would not impose an impact on capital stops [11]. With the deepening of research and changes in global economic environment, the role of capital account liberalization has gradually changed. It could increase the probability of sudden stops, and financial development would help to alleviate the influence of capital account [12]. However, capital control could effectively reduce the probability of sudden stops, and economies with strict capital control need more foreign exchange reserves to prevent sudden stops [13]. In recent years, with the transfer of economic policies in developed countries and the escalation of global economic and trade frictions, global economic policy uncertainty has been rising. Changing the direction and scale of international capital flows through investor expectations, global economic policy uncertainty has gradually become an important factor that causes episodes of extreme international capital inflows.

To sum up, the existing literature is of great reference value for this study, but there are still shortcomings. First of all, macro uncertainties have not been considered. Under the background of the complicated and changeable international economic environment, traditional push factors and pull factors can no longer fully explain the changes of international capital flows, and uncertainties should be incorporated into the research framework. Secondly, in terms of heterogeneity test, the existing literature mostly focuses on the differences between developed economies and emerging and developing economies, and fails to further analyze on other attributes of a certain type of economy. Finally, researchers mainly focus on direct effect model and mediating effect model, and rarely discuss whether the impact of global economic policy uncertainty on surges and stops changes with its rise.

Contributions of this paper may lie in the following aspects: firstly, we focus the external factor that influences the episodes of extreme international capital inflows (surges and stops) on global economic policy uncertainty, rather than variables such as interest rates and global liquidity mentioned in the existing literature. Global economic policy uncertainty is a novel influencing factor. Secondly, the existing relevant literature is limited to comparing the similarities and differences of influencing factors between advanced and emerging and developing economies. This paper, however, only selects emerging and developing economies as research objects, and discusses the roles of variables such as economic growth and financial development in the process of global economic policy uncertainty affecting episodes of extreme international capital inflows. The research is helpful to deepen the previous achievements. Thirdly, this paper discusses the changes in the impact of global economic policy uncertainty on episodes of extreme international capital inflows as global economic policy uncertainty rises. The relevant conclusions can not only provide theoretical guidance for deepening domestic financial reform and preventing systemic risks, but also have certain reference value to emerging and developing economies to strengthen capital flow management, prevent episodes of extreme international capital inflows risks and formulate more targeted policies.

The remainder of this paper is structured as follows. Section 2 is theoretical analysis. Section 3 introduces the empirical method and measurements of variables. Section 4 discusses the empirical results. Section 5 is extensive analysis.

## 2. Theoretical analysis

Early research points out that uncertainty is a risk composed of various factors that can hardly be analyzed, calculated and foreseen accurately [14]. And economic policy uncertainty refers to the risk caused by the uncertainty of economic policies formulated by the government in future direction and intensity [15, 16].

According to the real option theory, the rising of economic policy uncertainty will lead to the unpredictability of investment prospects, and the irreversibility of capital will force investors to choose to “wait and see”. Therefore, the investors’ willingness and confidence to take risks will change due to economic policy uncertainty. In order to avoid risk exposure caused by the rising of global economic policy uncertainty, international investors are prone to investment activities in emerging and developing economies with relatively stable macro-economy, which will lead to an increase in international capital inflows, improve the probability of surges and reduce the probability of stops. On one hand, the preference for safe assets drives international capital to flow into emerging and developing economies, which can be referred to the safe asset transfer effect [17]. Compared with emerging and developing economies, the financial systems of developed economies are relatively open and more vulnerable to the rising of global economic policy uncertainty. Under this circumstance, the instability of earnings in developed markets drives international capital to flow into the emerging and developing economies. On the other hand, increased risks in developed economies have also driven international capital to flow into emerging and developing economies, which can be called the global portfolio rebalancing effect [17]. As far as the composition is concerned, the global economic policy uncertainty is mainly dominated by developed economies, and the rising of global economic policy uncertainty means macroeconomic instability in developed economies, which further strengthens the preference and investment stickiness of risk-averse investors to the assets of emerging and developing economies. With the increase of global economic policy uncertainty, the demand of foreign investors for assets in emerging and developing economies will rise sharply, while the demand of foreign investors for assets in developed economies will rise slightly. Therefore:

H: The rising of global economic policy uncertainty can increase the probability of surges and decrease the probability of stops in emerging and developing economies.

## 3. Method and measurements

### 3.1 Empirical method

In this paper, the impact of global economic policy uncertainty on episodes of extreme international capital inflows is analyzed by building a panel regression model. As the dependent variable is a dummy variable, the panel Probit model is adopted for analysis. The specific model is as follows:

$$P(Surges_{i,t}=1)=F(\alpha +A^{\prime }Gepu_{t}+B^{\prime }X_{i,t})+\upsilon_{i,t}$$ (1)

$$P(Stops_{i,t}=1)=F(\beta +C^{\prime }Gepu_{t}+D^{\prime }X_{i,t})+\vartheta_{i,t}$$ (2)

Where *Surges*_*i*,*t*_ = 1 indicates an episode dummy variable that takes the value of 1 if economy *i* is experiencing a surge in period *t* [18]. Similarly, *Stops*_*i*,*t*_ = 1 indicates an episode dummy variable that takes the value of 1 if economy *i* is experiencing a stop in period *t*. *Gepu*_*t*_ refers to the global economic policy uncertainty, *X*_*i*,*t*_ is a vector of control variables, and *υ* and *ϑ* are random error terms of Eq (1) and Eq (2), respectively.

### 3.2 Measurements of variables and data sources

#### 3.2.1. Measurements of extreme international capital inflows episodes

Extreme international capital flows include extreme international capital inflows and extreme international capital outflows [1, 18]. Extreme international capital inflows episodes are driven by foreign investors and can be divided into surges and stops, and extreme international capital outflows episodes are driven by domestic investors and can be divided into flight and retrenchment [18]. This paper follows this classification and selects extreme international capital inflows episodes as the subject of study.

As for the measurement of extreme international capital inflows episodes, early studies mostly focused on the sharp decrease of net capital inflows [18, 19]. With the deepening of research, the net capital inflows can no longer reflect behaviors of investment and financing of domestic and foreign investors, and studies on capital inflow episodes have gradually relied on proxies for gross capital inflows [1, 18]. This paper also focuses on gross international capital inflows (proportion in GDP). The relevant data come from the IMF Database.

Mainly driven by foreign investors, extreme international capital inflows episodes are dummy variables whose proxies can be calculated by three approaches: approach of threshold [11, 20, 21], approach of standard deviation [18, 22], approach of combination of above two methods [12, 23]. The problem with these approaches, however, is that they rely on threshold criteria which specifies the period above the threshold as a label for “extreme episodes” [24]. A three-state Markov-switching model used to identify periods of extreme net flows can compensate for the shortcomings of above methods [24, 25]. This method can also be applied to gross capital inflows and identify episodes of extreme international capital inflows more accurately.

Given the dynamic nature of international capital flows, we also believe that international capital inflows follow a process of regime switching [25]. They refer to normal periods and two extreme periods. According to the characteristics of international capital inflows, we take the international capital inflows in normal periods as a reference, and define extreme periods when international capital flows are higher than them as surges, and extreme periods when international capital flows are lower than them as stops. Our definitions of extreme international capital inflows episodes closely follow the statistical criteria used by the above literature which adapted the three-state Markov-switching model. That is, states are defined by switches in the mean of the capital inflows, and the dynamics of extreme international capital inflows are qualitatively different from those of normal international capital inflows [24, 25]. We can obtain probabilistic classification of observations in different states through parametric estimates. These probabilities are estimated based on international capital inflows data.

#### 3.2.2 Measurements of global economic policy uncertainty

After the outbreak of financial crisis in 2008, researchers have constructed or measured the uncertainty from different perspectives. For example, they construct the uncertainty index based on stochastic volatility of real interest rate, political election, predictive index and media analysis [26–29]. Based on newspaper coverage frequency, Baker, Bloom and Davis have built indexes of economic policy uncertainty which could well depict the economic policy uncertainty and have been widely applied [28].

According to Baker et al., the global economic policy uncertainty index is a GDP-weighted average of national EPU indexes for 21 countries: Australia, Brazil, Canada, Chile, China, Colombia, France, Germany, Greece, India, Ireland, Italy, Japan, Mexico, the Netherlands, Russia, South Korea, Spain, Sweden, the United Kingdom, and the United States [28]. This paper, however, only selects emerging and developing economies as research objects. To more accurately describe the economic policy uncertainty caused by advanced economies, we excluded a sample of emerging and developing economies from the 21 economies mentioned above. The Global economic policy uncertainty Index (*Gepu*) in this paper is a GDP-weighted average of national EPU indexes for 14 major advanced economies: Australia, Canada, France, Germany, Greece, Ireland, Italy, Japan, the Netherlands, South Korea, Spain, Sweden, the United Kingdom, and the United States. The relevant data come from the website of Economic Policy Uncertainty.

#### 3.2.3 Measurements of control variables

Following the existing literature [4, 12, 30–32], this paper selects control variables from both global and domestic aspects. The definitions and data sources are shown in Table 1.

**Table 1:** Control variables.

	Variable Name	Definition	Data Source
Global Variables	Global Liquidity (*Gliq)*	Broad Money (% of GDP)	World Bank Database
	Global Interest Rate (*Gint*)	US Federal Funds Rate	Wind Database
	Global Risk (*Vix*)	S & P 500 Volatility Index	Wind Database
Domestic Variables	Capital Account Liberalization (*Ka*)	The Sum of International Capital Inflows and International Capital Outflows (% of GDP)	IMF Database
	Financial Development (*Fd*)	Domestic Credit to Private Sector (% of GDP)	World Bank Database
	Economic Growth (*Gdp*)	GDP Per Capita Growth (annual %)	World Bank Database
	Exchange Rate (*Reer*)	Real Effective Exchange Rate Index	World Bank Database and BIS Database
	Trade Opening (*Topen*)	Total Trade (% of GDP)	IFS Database
	Political Stability (*P*)	Political Stability and Absence of Violence/Terrorism	WGI Database

Notes: Table 1 describes the names, definitions, and data sources of control variables.

### 3.3 Descriptive statistics

Based on data availability, we select annual data for 31 emerging and developing economies in the period 2000–2020. The descriptive statistics of variables are shown in Table 2. During the sample period, there are 165 surges and 184 stops. The summary of international capital inflows in each state is shown in Table 3.

**Table 2:** Descriptive statistics of variables.

Variable	Observation	Mean	SD	Min	Max
*Surges*	651	0.2535	0.4353	0.0000	1.0000
*Stops*	651	0.2826	0.4506	0.0000	1.0000
*Gepu*	651	100.9317	31.8319	48.9608	163.2300
*Gliq*	651	109.0536	11.8357	95.5898	134.3277
*Gint*	651	1.4295	1.6622	0.0400	5.4100
*Vix*	651	19.8100	6.8409	11.0400	40.0000
*Ka*	651	9.1445	15.1741	-78.5357	189.9460
*Fd*	651	52.5803	36.3348	7.1252	182.4326
*Gdp*	651	2.6084	4.2471	-19.6347	14.6959
*Reer*	651	96.7943	16.9515	54.0582	276.3800
*Topen*	651	56.1456	63.3817	0.0188	493.4646
*P*	651	-0.3883	0.8057	-4.4320	1.8054

Notes: Table 2 reports descriptive statistics of variables, including observations, means, standard deviations, maximums, and minimum values.

**Table 3:** Summary of international capital inflows in each state.

States	Occurrence	Mean	Standard Deviation
Surge	165	11.9058	11.9996
Normal	302	5.5330	5.6120
Stop	184	1.5258	6.5492
Total	651		

Notes: Table 3 reports summary of international capital inflows in each state.

The 31 emerging and developing economies mentioned are as follows: Argentina, Armenia, Bolivia, Brazil, Chile, China, Colombia, Dominica, Fiji, Grenada, Hungary, India, Indonesia, Malaysia, Mexico, Moldova, Nicaragua, Nigeria, Paraguay, Peru, Philippines, Poland, Romania, Russian, South Africa, Thailand, Togo, Tunisia, Turkey, Uganda, and Ukraine.

**Fig 1:**
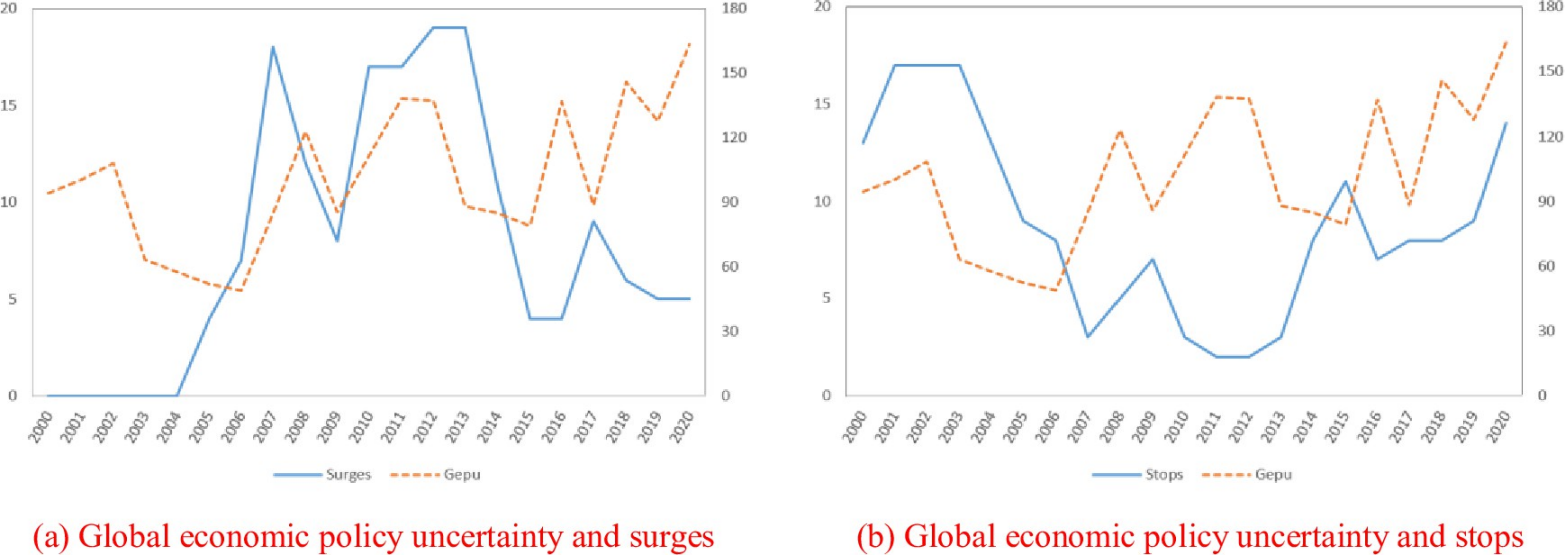
Global economic policy uncertainty and episodes of surges and stops. (a) Global economic policy uncertainty and surges; (b) Global economic policy uncertainty and stops. Notes: Left panel shows the relationship between global economic policy uncertainty and surges, where the blue solid line refers to the number of surges. Right panel shows the relationship between global economic policy uncertainty and stops, where the blue solid line refers to the number of stops. The orange dotted line refers to global economic policy uncertainty index.

Fig 1 shows the relationship between global economic policy uncertainty and episodes. In this figure, x-axis represents the year, y-axis (left) represents number of economies that experienced surges or stops in the sample every year and y-axis (right) represents the global economic policy uncertainty index. According to Fig 1, global economic policy uncertainty (*Gepu*) and episodes of surges have the same trend, while the trend of global economic policy uncertainty (*Gepu*) and episodes of stops is opposite. Whether episodes of extreme international capital inflows are significantly affected by global economic policy uncertainty still needs further empirical test.

## 4. Empirical result analysis

### 4.1 Baseline result

Our analyses focus on the impact of global economic policy uncertainty on episodes of surges and stops. The benchmark regression results are shown in Table 4. As can be seen from M (1), M (3) and M (5) in Table 4, the coefficients of global economic policy uncertainty are significantly positive, which indicates that the rising of external uncertainty can improve the probability of surges in emerging and developing economies. According to M (2), M (4) and M (6) in Table 4, the coefficients of global economic policy uncertainty are significantly negative, which means that the rising of external uncertainty can effectively reduce the probability of stops in emerging and developing economies. Therefore, hypothesis H has been validated. In terms of the composition, the global economic policy uncertainty is dominated by developed economies, and its rising stands for the unpredictability of earnings in the developed markets. On one hand, the domestic macroeconomic stability of developed economies has decreased with the rising of economic policy uncertainty, which will prompt risk-averse investors to prefer the assets of emerging and developing economies (safe asset transfer effect). On the other hand, the instability of excess earnings in developed economies will force investors to adjust their asset portfolios worldwide, while the higher investment returns in emerging and developing economies will attract international capital inflows easily (global portfolio rebalancing). Therefore, under the combined action of safe asset transfer effect and global portfolio rebalancing [17], the rising of global economic policy uncertainty will drive capital flows to emerging and developing economies, increase the probability of surges and decrease the probability of stops.

**Table 4:** Impact of global economic policy uncertainty on episodes of surges and stops.

	*Surges*	*Stops*	*Surges*	*Stops*	*Surges*	*Stops*
	M (1)	M (2)	M (3)	M (4)	M (5)	M (6)
*Gepu*	0.0051***	-0.0030*	0.0080***	-0.0062**	0.0068**	-0.0064**
	(3.12)	(-1.80)	(2.70)	(-2.12)	(2.08)	(-2.05)
*Gliq*			-0.0195***	0.0091	-0.0086	0.0020
			(-2.58)	(1.16)	(-0.92)	(0.23)
*Gint*			-0.1742***	0.0488	-0.1609***	0.0229
			(-4.07)	(1.37)	(-3.34)	(0.61)
*Vix*			-0.0283**	0.0228**	-0.0146	0.0124
			(-2.47)	(2.18)	(-1.24)	(1.09)
*Ka*					0.0700***	-0.0540***
					(4.87)	(-3.08)
*Fd*					0.0066***	-0.0002
					(3.57)	(-0.14)
*Gdp*					0.0612***	-0.0471***
					(3.52)	(-3.10)
*Reer*					0.0026	-0.0062*
					(0.65)	(-1.79)
*Topen*					-0.0003	-0.0019
					(-0.39)	(-1.61)
*P*					-0.6270***	0.3675***
					(-6.27)	(4.12)
*Constant*	-1.1829***	-0.2707	1.4388*	-1.4687*	-1.7034	0.9347
	(-6.75)	(-1.54)	(1.83)	(-1.78)	(-1.62)	(0.97)
Observations	651	651	651	651	651	651
Pseudo R^2^	0.0120	0.0045	0.0407	0.0115	0.2520	0.1363

**Notes**: Table 4 reports empirical results of benchmark regression. Values in parentheses are robust z-statistics.

***, **, and * denote significance at the 1%, 5% and 10% levels, respectively.

From the perspective of risks and benefits, the market risk in developed economies will increase with the rising of global economic policy uncertainty, which will drive changes of international capital flows direction under the given earnings. When the global economic policy uncertainty is expected to rise for a long time in the future, international investors are more prone to long-term investment activities in economies with a stable macroeconomic environment. Besides, the high economic growth rate and expected return in emerging and developing economies are extremely attractive for direct investment. Due to capital control cost, the rising of global economic policy uncertainty will also prompt investors who have already held long-term assets in the capital receiving country to hold the assets in emerging and developing economies more firmly.

### 4.2 Heterogeneity test

As mentioned above, the impact of global economic policy uncertainty on surges and stops in emerging and developing economies is discussed on the whole. However, demands of investors will vary according to the level of economic development, the degree of openness, etc., which will have different impacts on the direction and scale of international capital inflows.

Hence, this paper examines heterogeneity from four aspects: economic growth, financial development, economic openness, and global liquidity, which can help to explore their roles in the process of global economic policy uncertainty affecting episodes of extreme international capital inflows. It should be pointed out that we choose the economic globalization sub-indicator of the KOF Globalization Index to measure the degree of economic openness [33–35].

In addition, we divided the sample into higher group and lower group by a 50% quantile of each variable. The heterogeneity test results are shown in Tables 5 to 8.

**Table 5:** Heterogeneity test results of economic growth.

	Higher Economic Growth		Lower Economic Growth	
	*Surges*	*Stops*	*Surges*	*Stops*
	M (1)	M (2)	M (3)	M (4)
*Gepu*	0.0131***	-0.0134**	0.0013	-0.0049
	(2.60)	(-2.25)	(0.30)	(-1.18)
*Gliq*	-0.0191	0.0169	0.0010	0.0045
	(-1.28)	(1.10)	(0.08)	(0.38)
*Gint*	-0.0895	0.0078	-0.2886***	0.0585
	(-1.47)	(0.13)	(-2.60)	(1.07)
*Vix*	-0.0231	0.0140	-0.0062	0.0227
	(-1.37)	(0.69)	(-0.35)	(1.50)
*Ka*	0.0866***	-0.0781***	0.0558***	-0.0438**
	(6.67)	(-4.56)	(3.05)	(-2.21)
*Fd*	0.0071***	-0.0075***	0.0055**	0.0040*
	(2.66)	(-2.71)	(2.16)	(1.66)
*Gdp*	0.0484	0.0090	0.0441	-0.0533**
	(1.20)	(0.25)	(1.58)	(-2.30)
*Reer*	0.0121*	-0.0143*	-0.0010	-0.0019
	(1.70)	(-1.89)	(-0.17)	(-0.53)
*Topen*	-0.0006	-0.0099**	-0.0002	0.0004
	(-0.54)	(-2.03)	(-0.11)	(0.21)
*P*	-0.8873***	0.5789***	-0.3812***	0.3331***
	(-6.57)	(4.08)	(-3.08)	(2.68)
*Constant*	-2.2393	1.3619	-1.6419	-0.5956
	(-1.37)	(0.79)	(-1.08)	(-0.47)
Observations	325	325	326	326
Pseudo R^2^	0.2964	0.2573	0.2298	0.1149

**Notes**: Table 5 reports heterogeneity test results of economic growth. Values in parentheses are robust z-statistics.

***, **, and * denote significance at the 1%, 5% and 10% levels, respectively.

**Table 6:** Heterogeneity test results of financial development.

	Higher Financial Development		Lower Financial Development	
	*Surges*	*Stops*	*Surges*	*Stops*
	M (1)	M (2)	M (3)	M (4)
*Gepu*	0.0085**	-0.0082*	0.0048	-0.0082
	(2.08)	(-1.82)	(0.76)	(-1.39)
*Gliq*	-0.0284**	0.0189	-0.0106	-0.0088
	(-2.06)	(1.35)	(-0.64)	(-0.64)
*Gint*	-0.2032***	0.0492	-0.0852	-0.0496
	(-3.01)	(0.90)	(-1.22)	(-0.89)
*Vix*	-0.0318**	0.0229	-0.0031	0.0110
	(-1.99)	(1.42)	(-0.16)	(0.55)
*Ka*	0.0519***	-0.0450***	0.1630***	-0.1888***
	(4.26)	(-2.85)	(5.51)	(-6.32)
*Fd*	0.0050**	-0.0051*	0.0156	0.0137
	(1.98)	(-1.96)	(1.25)	(1.37)
*Gdp*	0.0423*	-0.0861***	0.0426*	0.0509*
	(1.84)	(-3.58)	(1.81)	(1.68)
*Reer*	0.0012	-0.0046	0.0073	-0.0007
	(0.20)	(-0.79)	(1.39)	(-0.13)
*Topen*	-0.0010	-0.0012	0.0089	-0.0126**
	(-1.23)	(-1.02)	(1.34)	(-2.01)
*P*	-0.7603***	0.5151***	-0.6700***	0.1382
	(-4.05)	(2.85)	(-4.48)	(1.21)
*Constant*	1.2580	-0.7427	-3.4159**	1.8353
	(0.77)	(-0.45)	(-1.96)	(1.23)
Observations	325	325	326	326
Pseudo R^2^	0.2275	0.1915	0.4221	0.3285

**Notes**: Table 6 reports heterogeneity test results of financial development. Values in parentheses are robust z-statistics.

***, **, and * denote significance at the 1%, 5% and 10% levels, respectively.

**Table 7:** Heterogeneity test results of economic openness.

	Higher Economic Openness		Lower Economic Openness	
	*Surges*	*Stops*	*Surges*	*Stops*
	M (1)	M (2)	M (3)	M (4)
*Gepu*	0.0096**	-0.0075*	0.0044	-0.0065
	(2.10)	(-1.80)	(0.92)	(-1.05)
*Gliq*	-0.0328**	0.0117	0.0178	-0.0203
	(-2.49)	(1.00)	(1.24)	(-1.19)
*Gint*	-0.1898***	-0.0398	-0.0951	0.0687
	(-2.81)	(-0.74)	(-1.33)	(1.15)
*Vix*	-0.0192	0.0168	-0.0127	0.0047
	(-1.19)	(1.08)	(-0.73)	(0.23)
*Ka*	0.0671***	-0.0448**	0.0781***	-0.1359***
	(3.21)	(-2.50)	(5.79)	(-4.12)
*Fd*	0.0052**	0.0002	0.0094***	0.0013
	(2.14)	(0.10)	(2.81)	(0.41)
*Gdp*	0.0263	-0.0323	0.0872***	-0.0450
	(1.18)	(-1.62)	(2.90)	(-1.60)
*Reer*	0.0118*	-0.0043	-0.0091	-0.0024
	(1.89)	(-0.70)	(-1.39)	(-0.53)
*Topen*	-0.0012	-0.0027*	0.0070	-0.0064
	(-1.27)	(-1.91)	(1.54)	(-0.99)
*P*	-0.6028***	0.4438***	-0.8912***	0.0380
	(-4.11)	(3.45)	(-5.58)	(0.26)
*Constant*	0.2627	-0.1071	-4.1118**	2.9527*
	(0.18)	(-0.08)	(-2.49)	(1.71)
Observations	341	341	310	310
Pseudo R^2^	0.2742	0.1386	0.2825	0.2954

**Notes**: Table 7 reports heterogeneity test results of economic openness. Values in parentheses are robust z-statistics.

***, **, and * denote significance at the 1%, 5% and 10% levels, respectively.

**Table 8:** Heterogeneity test results of global liquidity.

	Higher Global Liquidity		Lower Global Liquidity	
	*Surges*	*Stops*	*Surges*	*Stops*
	M (1)	M (2)	M (3)	M (4)
*Gepu*	0.0075*	-0.0124***	-0.0010	0.0019
	(1.96)	(-2.88)	(-0.09)	(0.21)
*Gliq*	-0.0661***	0.0600***	0.1251***	-0.0849**
	(-3.24)	(3.36)	(2.84)	(-2.30)
*Gint*	-0.0838	0.0575	0.0870*	-0.0423
	(-0.57)	(0.47)	(1.79)	(-0.79)
*Vix*	-0.0222	0.0189	0.0215	-0.0109
	(-0.99)	(0.85)	(0.89)	(-0.46)
*Ka*	0.0562***	-0.0285**	0.0793***	-0.1480***
	(3.15)	(-2.45)	(6.11)	(-5.75)
*Fd*	0.0058***	-0.0011	0.0097***	0.0015
	(2.67)	(-0.46)	(3.16)	(0.56)
*Gdp*	0.0208	-0.0571**	0.1127***	0.0213
	(0.84)	(-2.19)	(3.97)	(0.84)
*Reer*	-0.0052	-0.0077	0.0102*	0.0040
	(-0.83)	(-1.32)	(1.94)	(0.87)
*Topen*	-0.0004	0.0024	-0.0014	-0.0026*
	(-0.25)	(1.31)	(-1.32)	(-1.72)
*P*	-0.7498***	0.7313***	-0.6720***	0.1803
	(-5.97)	(5.63)	(-4.71)	(1.58)
*Constant*	6.1323**	-5.7290***	-16.9024***	8.5171**
	(2.43)	(-2.72)	(-4.17)	(2.57)
Observations	310	310	341	341
Pseudo R^2^	0.2444	0.2009	0.3840	0.2932

**Notes**: Table 8 reports heterogeneity test results of global liquidity. Values in parentheses are robust z-statistics.

***, **, and * denote significance at the 1%, 5% and 10% levels, respectively.

#### 4.2.1 Heterogeneity of economic growth

According to Table 5, the impact of global economic policy uncertainty on surges and stops are significant in economies with higher economic growth. However, in economies with lower economic growth, the impact of global economic policy uncertainty is no longer significant. These results show that economic growth of an economy is exactly an important reference factor for investors to select assets. When allocating assets in emerging and developing economies, foreign investors are more inclined to choose the assets from economies with relatively high economic growth, which will lead to a sharp increase in demand for these assets. However, the demand of foreign investors for assets in lower economic growth economies increases slightly. These results may be caused by the following reasons. Economies with relatively high economic growth tend to provide a more stable macroeconomic environment that can enhance the confidence and sense of security of fund holders and easily attract international capital inflows. In contrast, economies with relatively low economic growth may lose their competitiveness due to poor economic fundamentals.

#### 4.2.2 Heterogeneity of financial development

According to Table 6, the impact of global economic policy uncertainty on surges and stops are significant in economies with higher financial development. However, in economies with lower financial development, the impact of global economic policy uncertainty is no longer significant. These results show that financial development of an economy is also an important reference factor for investors to select assets. On one hand, better financial development means that the economy has abundant financial instruments and efficient intermediaries, which can change the traditional “deposit-investment” cycle mode and improve the efficiency of social capital distribution. On the other hand, the improvement of financial development can not only directly broaden external financing channels, reduce domestic financing costs and liquidity risks, but also indirectly boost economic growth. Therefore, the better financial development can stabilize the inflows of international capital.

#### 4.2.3 Heterogeneity of economic openness

According to Table 7, the impact of global economic policy uncertainty on surges and stops are significant in economies with higher economic openness. However, in economies with lower economic openness, the impact of global economic policy uncertainty is no longer significant. These results show that economic openness of an economy is also an important reference factor for investors to select assets. The lower the degree of economic openness is, the higher the cost of domestic investment by international capital. In the principle of profit maximization, international investors are more willing to hold the assets of countries with a high degree of economic openness. Therefore, relatively high economic openness will promote international capital inflows because of the low cost. In addition, the relatively high degree of economic openness means that the economy has more economic communications with other countries and has strong development potential. Higher economic openness also means greater efficiency in domestic resource allocation, which helps to improve investment returns.

#### 4.2.4 Heterogeneity of global liquidity

According to Table 8, the impact of global economic policy uncertainty on surges and stops are significant when global liquidity is relatively high. However, the impact of global economic policy uncertainty is not significant when global liquidity is relatively low. These results show that global liquidity plays an important role when global economic policy uncertainty affects surges and stops. Higher global liquidity means that investors hold more liquid assets. Sufficient liquidity will accelerate the flow of international capital into emerging and developing economies when the global economic policy uncertainty rises.

### 4.3 Robustness test

We use five methods to verify the robustness of the basic test. The first is to replace the measurement of global economic policy uncertainty. We calculate the arithmetic average (*Gepu*1) of economic policy uncertainty indexes of the 14 major developed economies mentioned above. The second is to replace *Gepu* with *Gepu*2 which is a GDP-weighted average of national EPU indexes for 4 major developed economies: the United States, Japan, the United Kingdom, and European Union. This method narrows the measurement range of global economic policy uncertainty in the previous empirical analysis. The third is to exclude special data of 2008 and 2020 in which there are emergencies affecting international capital flows. The fourth is to change the empirical method, that is, to replace the Probit model with the Logit model. The fifth is to add the sovereign debt risk (*Debt*) and exchange rate regime (*Re*) to the control variables that can overcome the errors caused by omitted variables.

The results reported in Tables 9 and 10 show that global economic policy uncertainty still has a positive impact on surges and a negative impact on stops, indicating that the previous empirical results are basically robust.

**Table 9:** Robustness test results of another proxy variable.

	the First Proxy Variable		the Second Proxy Variable	
	*Surges*	*Stops*	*Surges*	*Stops*
	M (1)	M (2)	M (3)	M (4)
*Gepu*1	0.0077***	-0.0088***		
	(2.85)	(-3.17)		
*Gepu*2			0.0043**	-0.0037*
			(1.98)	(-1.78)
*Gliq*	-0.0159	0.0124	-0.0116	0.0034
	(-1.55)	(1.39)	(-1.10)	(0.35)
*Gint*	-0.1360***	-0.0087	-0.1706***	0.0279
	(-2.77)	(-0.22)	(-3.53)	(0.74)
*Vix*	-0.0158	0.0152	-0.0110	0.0077
	(-1.48)	(1.44)	(-1.01)	(0.74)
*Ka*	0.0690***	-0.0529***	0.0700***	-0.0539***
	(4.87)	(-3.07)	(4.85)	(-3.07)
*Fd*	0.0067***	-0.0003	0.0066***	-0.0002
	(3.59)	(-0.19)	(3.57)	(-0.14)
*Gdp*	0.0549***	-0.0396***	0.0608***	-0.0464***
	(3.10)	(-2.62)	(3.48)	(-3.05)
*Reer*	0.0025	-0.0060*	0.0028	-0.0063*
	(0.60)	(-1.75)	(0.68)	(-1.80)
*Topen*	-0.0004	-0.0018	-0.0003	-0.0020
	(-0.52)	(-1.48)	(-0.37)	(-1.61)
*P*	-0.6261***	0.3634***	-0.6278***	0.3678***
	(-6.30)	(4.08)	(-6.28)	(4.14)
*Constant*	-1.1904	0.2012	-1.3632	0.7539
	(-1.07)	(0.21)	(-1.18)	(0.70)
Observations	651	651	651	651
Pseudo R^2^	0.2572	0.1452	0.2513	0.1351

**Notes**: Table 9 reports robustness test results of another proxy variable. Values in parentheses are robust z-statistics.

***, **, and * denote significance at the 1%, 5% and 10% levels, respectively.

**Table 10:** Robustness test results of excluding special data, Logit model and adding two variables.

	Excluding Special Data		Logit Model		Adding Variable *Debt* and *Re*	
	*Surges*	*Stops*	*Surges*	*Stops*	*Surges*	*Stops*
	M (1)	M (2)	M (3)	M (4)	M (5)	M (6)
*Gepu*	0.0075**	-0.0084**	0.0112**	-0.0099*	0.0059*	-0.0068**
	(2.27)	(-2.57)	(1.96)	(-1.79)	(1.69)	(-1.98)
*Gliq*	-0.0080	0.0011	-0.0131	0.0029	-0.0115	0.0016
	(-0.83)	(0.12)	(-0.79)	(0.19)	(-1.15)	(0.17)
*Gint*	-0.1563***	0.0023	-0.1563***	0.0268	-0.1563***	0.0265
	(-3.24)	(0.06)	(-3.04)	(0.41)	(-3.20)	(0.64)
*Vix*	-0.0216	0.0255*	-0.0241	0.0182	-0.0167	0.0135
	(-1.34)	(1.73)	(-1.18)	(0.91)	(-1.32)	(1.10)
*Ka*	-0.1563***	-0.1563***	-0.1563***	-0.1563***	-0.1563***	-0.0545**
	(4.61)	(-2.74)	(5.55)	(-4.09)	(3.58)	(-2.44)
*Fd*	-0.1563***	-0.0005	-0.1563***	0.0004	-0.1563***	-0.0014
	(3.56)	(-0.26)	(3.24)	(0.13)	(3.08)	(-0.69)
*Gdp*	-0.1563***	-0.0259	-0.1563***	-0.0676**	-0.1563***	-0.1563***
	(3.02)	(-1.51)	(3.14)	(-2.55)	(3.44)	(-2.62)
*Reer*	0.0011	-0.0053	0.0034	-0.0094	0.0041	-0.0063
	(0.25)	(-1.46)	(0.48)	(-1.55)	(0.98)	(-1.48)
*Topen*	-0.0003	-0.0027*	-0.0008	-0.0036	0.0058*	-0.0034
	(-0.36)	(-1.84)	(-0.55)	(-1.51)	(1.95)	(-1.19)
*P*	-0.1563***	-0.1563***	-0.1563***	-0.1563***	-0.1563***	-0.1563***
	(-5.95)	(3.85)	(-6.30)	(4.05)	(-3.95)	(3.43)
*Debt*					0.0058	-0.1563***
					(1.42)	(-3.02)
*Re*					-0.1563***	-0.0020
					(3.63)	(-0.09)
*Constant*	-1.5315	0.8525	-2.9850	1.6665	-2.8003**	1.4148
	(-1.38)	(0.85)	(-1.60)	(1.01)	(-2.15)	(1.25)
Observations	589	589	651	651	546	546
Pseudo R^2^	0.2425	0.1308	0.2522	0.1503	0.2607	0.1336

**Notes**: Table 10 reports robustness test results of excluding special data, Logit model and adding two variables. Values in parentheses are robust z-statistics.

***, **, and * denote significance at the 1%, 5% and 10% levels, respectively.

## 5. Extensive analysis

### 5.1 Discussion on the impact of *Gepu* to varying degrees

According to the conclusions of basic test, the global economic policy uncertainty will increase the probability of surges and decrease the probability of stops in emerging and developing economies. Another question worth thinking about is whether the impact of global economic policy uncertainty on surges and stops changes with its rise. What characteristics are presented? In view of this, this paper further explores the impact of global economic policy uncertainty to varying degrees. We have chosen five representative quantiles of global economic policy uncertainty for discussion. They are not higher than the 30% quantile, not higher than the 40% quantile, not higher than the 50% quantile, not higher than the 60% quantile and not higher than the 70% quantile, respectively. The regression results are shown in Tables 11 and 12.

**Table 11:** Impact of the *Gepu* on surges to varying degrees.

	≦ 30% quantile	≦ 40% quantile	≦ 50% quantile	≦ 60% quantile	≦ 70% quantile
	*Surges*	*Surges*	*Surges*	*Surges*	*Surges*
	M (1)	M (2)	M (3)	M (4)	M (5)
*Gepu*	0.3186***	0.1050***	0.0630***	0.0113	0.0202***
	(2.58)	(6.70)	(5.12)	(1.36)	(3.24)
*Gliq*	0.0462	0.0382	-0.0218	0.0196	0.0103
	(1.07)	(1.61)	(-1.10)	(1.64)	(0.93)
*Gint*	1.2294***	0.6359***	0.2113**	0.0057	-0.0106
	(3.56)	(4.84)	(2.49)	(0.12)	(-0.21)
*Vix*	-0.7122**	-0.0994***	-0.0180	-0.0345	-0.0560***
	(-1.97)	(-2.64)	(-0.57)	(-1.36)	(-2.63)
*Ka*	0.0505***	0.0711***	0.0486***	0.0548***	0.0560***
	(3.71)	(5.16)	(3.10)	(3.35)	(3.39)
*Fd*	0.0125***	0.0132***	0.0116***	0.0090***	0.0088***
	(3.39)	(4.12)	(4.27)	(3.65)	(3.67)
*Gdp*	0.0923***	0.0276	0.0366	0.0524**	0.0555**
	(2.61)	(0.97)	(1.43)	(2.11)	(2.33)
*Reer*	-0.0053	-0.0063	0.0043	0.0057	0.0056
	(-0.50)	(-0.68)	(0.56)	(1.29)	(1.30)
*Topen*	-0.0042*	-0.0038*	-0.0025	-0.0012	-0.0009
	(-1.93)	(-1.91)	(-1.48)	(-1.19)	(-0.99)
*P*	-0.8699***	-0.8914***	-0.7533***	-0.5945***	-0.5786***
	(-4.72)	(-5.75)	(-5.32)	(-4.59)	(-4.63)
*Constant*	-20.2713***	-13.1097***	-5.0482**	-5.1820***	-4.4805***
	(-3.56)	(-4.54)	(-2.41)	(-3.87)	(-3.44)
Observations	217	279	310	403	434
Pseudo R^2^	0.4286	0.4144	0.3098	0.2415	0.2633

**Notes**: Table 11 shows impact of the *Gepu* on surges to varying degrees. Values in parentheses are robust z-statistics.

***, **, and * denote significance at the 1%, 5% and 10% levels, respectively.

**Table 12:** Impact of the *Gepu* on stops to varying degrees.

	≦ 30% quantile	≦ 40% quantile	≦ 50% quantile	≦ 60% quantile	≦ 70% quantile
	*Stops*	*Stops*	*Stops*	*Stops*	*Stops*
	M (1)	M (2)	M (3)	M (4)	M (5)
*Gepu*	-0.0334	-0.0478***	-0.0346***	-0.0029	-0.0122**
	(-1.14)	(-3.36)	(-2.93)	(-0.36)	(-2.00)
*Gliq*	0.0177	0.0177	0.0150	-0.0153	-0.0076
	(0.72)	(0.97)	(0.92)	(-1.39)	(-0.74)
*Gint*	-0.1930**	-0.1809**	-0.1584**	-0.0566	-0.0477
	(-2.08)	(-2.14)	(-1.99)	(-1.22)	(-1.01)
*Vix*	0.0234	0.0619	0.0066	0.0150	0.0408**
	(0.25)	(1.56)	(0.23)	(0.61)	(1.99)
*Ka*	-0.0909***	-0.0831***	-0.0360*	-0.0419**	-0.0441**
	(-4.27)	(-4.63)	(-1.82)	(-2.07)	(-2.20)
*Fd*	0.0001	-0.0006	-0.0020	-0.0000	-0.0002
	(0.03)	(-0.23)	(-0.78)	(-0.01)	(-0.09)
*Gdp*	0.0471*	0.0037	-0.0237	-0.0217	-0.0231
	(1.67)	(0.15)	(-1.06)	(-1.15)	(-1.21)
*Reer*	-0.0031	-0.0047	-0.0095	-0.0029	-0.0032
	(-0.46)	(-0.70)	(-1.46)	(-0.75)	(-0.80)
*Topen*	-0.0053*	-0.0007	-0.0013	-0.0026*	-0.0026*
	(-1.90)	(-0.38)	(-0.79)	(-1.69)	(-1.69)
*P*	0.3535**	0.3179**	0.3302***	0.2205**	0.2334**
	(2.36)	(2.46)	(2.72)	(2.32)	(2.49)
*Constant*	1.0901	1.4641	2.0556	2.0781*	1.5456
	(0.46)	(0.79)	(1.21)	(1.68)	(1.29)
Observations	217	279	310	403	434
Pseudo R^2^	0.2019	0.1968	0.1189	0.0964	0.1161

**Notes**: Table 12 shows impact of the *Gepu* on stops to varying degrees. Values in parentheses are robust z-statistics.

***, **, and * denote significance at the 1%, 5% and 10% levels, respectively.

As can be seen from Tables 11 and 12, it has the greatest impact on surges when global economic policy uncertainty is not higher than the 30% quantile, and it has the greatest impact on stops when global economic policy uncertainty is not higher than the 40% quantile. That is, the impact of global economic policy uncertainty on surges and stops gradually declines as it rises. Global economic policy uncertainty does not have the greatest impact when it is at a high level and it has a larger impact at lower levels.

### 5.2 Exploration of extreme international capital inflows episodes in developed economies

The previous theoretical analysis points out that the global economic policy uncertainty will promote foreign investors to allocate more assets in emerging and developing economies, increase the probability of surges and decrease the probability of stops. In other words, the rising of global economic policy uncertainty means that the capital inflows of developed economies will decrease, which will further reduce the probability of surges. Given that, this paper analyzes the impact of global economic policy uncertainty on episodes of surges and stops in 21 developed economies. The test results are as shown in Table 13.

**Table 13:** Impact of *Gepu* on extreme international capital inflows episodes in developed economies.

	*Surges*	*Stops*
	M (1)	M (2)
*Gepu*	-0.0087*	0.0001
	(-1.85)	(0.04)
*Gliq*	0.0186	0.0002
	(1.61)	(0.02)
*Gint*	0.2299***	-0.1239**
	(4.50)	(-2.14)
*Vix*	0.0216	0.0190
	(1.25)	(1.26)
*Ka*	0.0046***	-0.0118**
	(5.98)	(-2.00)
*Fd*	0.0089***	-0.0012
	(4.82)	(-0.70)
*Gdp*	-0.0270	-0.0325
	(-1.03)	(-1.28)
*Reer*	-0.0101*	-0.0174**
	(-1.76)	(-2.39)
*Topen*	0.0000	-0.0000**
	(0.60)	(-2.07)
*P*	-0.2452**	0.3247***
	(-2.22)	(2.63)
*Constant*	-2.7981**	0.8473
	(-2.30)	(0.60)
Observations	441	441
Pseudo R^2^	0.2006	0.1634

**Notes**: Table 13 shows impact of *Gepu* on extreme international capital inflows episodes in developed economies. Values in parentheses are robust z-statistics.

***, **, and * denote significance at the 1%, 5% and 10% levels, respectively.

The 21 developed economies referred to are the following: Australia, Canada, Czech Republic, Denmark, Estonia, France, Germany, Iceland, Israel, Italy, South Korea, Latvia, Malta, the Netherlands, Norway, Slovenia, Spain, Sweden, Switzerland, the United Kingdom, and the United States.

As can be seen from M (1) in Table 13, the coefficient of global economic policy uncertainty is significantly negative, which indicates that the rising of external uncertainty will reduce the probability of surges in developed economies. Besides, M (2) in Table 13 show that the rising of global economic policy uncertainty does not have a significant impact on stops in developed economies. The empirical results have validated the previous theoretical analysis, that is, foreign investors are more inclined to allocate the assets in emerging and developing economies and reduce the asset allocation in developed economies as global economic policy uncertainty increases.

## 6. Conclusions and implications

Based on annual data for 31 emerging and developing economies in the period 2000–2020, this paper explores the impact of global economic policy uncertainty on extreme movements in capital inflows. The analysis indicates that, in general, the global economic policy uncertainty can significantly increase the probability of surges and decrease the probability of stops. Above effects are significant in the groups with higher economic growth, higher financial development, higher economic globalization and higher global liquidity. Further analyses show that the impact of global economic policy uncertainty on surges and stops gradually declines as it rises. In developed economies, global economic policy uncertainty has a negative impact on surges, which has validated the above conclusions to some extent.

Under the background of global financial markets integration, the conclusions mentioned above have important implications for policymakers to manage cross-border capital flows in emerging and developing economies.

Firstly, our findings suggest that an early monitoring system for global economic policy uncertainty should be established, paying close attention to policy adjustments in developed economies. The rising of global economic policy uncertainty has an opposite effect on episodes of surges and stops in emerging and developing economies. The reversal of its direction will impose a huge impact on emerging and developing economies. Therefore, it is necessary to pay close attention not only to the traditional push and pull factors, but also to the trend of global economic policy uncertainty and guard against the risk of extreme movements in capital inflows.

Secondly, our findings suggest that collaborative management among economies should be strengthened. Global economic policy uncertainty has gradually become an important factor affecting the extreme movements in capital inflows, as well as an external factor confronted by all emerging and developing economies. The policy adjustment of a single economy has little effect. Therefore, economies should strengthen communication, establish multilateral cooperation, and jointly and rationally exploit the impact of external uncertainty on extreme movements in capital inflows.

## Supporting information

S1 Data Research data. (XLS)
